# Native drivers of fish life history traits are lost during the invasion process

**DOI:** 10.1002/ece3.6521

**Published:** 2020-08-03

**Authors:** Rodolphe Elie Gozlan, Eva Záhorská, Emira Cherif, Takashi Asaeda, John Robert Britton, Cha‐Ho Chang, To Hong, Rafael Miranda, Jiří Musil, Meta Povz, Ali Serhan Tarkan, Elena Tricarico, Teodora Trichkova, Hugo Verreycken, Andrej Weiperth, Andrej Witkowski, Lluis Zamora, Irene Zweimueller, Yahui Zhao, Hamid Reza Esmaeili, Marine Combe

**Affiliations:** ^1^ ISEM UMR226 CNRS IRD EPHE Université de Montpellier Montpellier France; ^2^ Faculty of Natural Sciences Department of Ecology Comenius University Bratislava Slovakia; ^3^ Department of Environmental Science Saitama University Saitama Japan; ^4^ School of Conservation Sciences Bournemouth University Poole UK; ^5^ Department of Biological Science and Technology National Chiao Tung University Hsinchu Taiwan ROC; ^6^ Department of Agriculture and Aquaculture Tra Vinh University Tra Vinh Vietnam; ^7^ Department of Environmental Biology School of Sciences University of Navarra Pamplona Spain; ^8^ Department of Aquatic Ecology T.G.Masaryk. Water Research Institute Prague 6 Czech Republic; ^9^ Zavod Umbra Ljubljana Slovenia; ^10^ Faculty of Fisheries Muğla Sıtkı Koçman University Muğla Turkey; ^11^ Dip. Biologia Universita’ degli Studi di Firenze Firenze Italy; ^12^ Institute of Biodiversity and Ecosystem Research Bulgarian Academy of Sciences Sofia Bulgaria; ^13^ Research Institute for Nature and Forest (INBO) Dwersbos Linkebeek Belgium; ^14^ MTA Centre for Ecological Research Danube Research Institute Budapest Hungary; ^15^ Museum of Natural History Wrocław University Wrocław Poland; ^16^ Faculty of Sciences Institute of Aquatic Ecology University of Girona Girona Spain; ^17^ Faculty of Life Sciences Department of Freshwater Ecology University of Vienna Vienna Austria; ^18^ Institute of Zoology Chinese Academy of Sciences Beijing China; ^19^ Department of Biology College of Sciences Shiraz University Shiraz Iran

**Keywords:** ecological impact, fish, genetic, Global changes, phenotype, plasticity

## Abstract

Rapid adaptation to global change can counter vulnerability of species to population declines and extinction. Theoretically, under such circumstances both genetic variation and phenotypic plasticity can maintain population fitness, but empirical support for this is currently limited. Here, we aim to characterize the role of environmental and genetic diversity, and their prior evolutionary history (via haplogroup profiles) in shaping patterns of life history traits during biological invasion. Data were derived from both genetic and life history traits including a morphological analysis of 29 native and invasive populations of topmouth gudgeon *Pseudorasbora parva* coupled with climatic variables from each location. General additive models were constructed to explain distribution of somatic growth rate (SGR) data across native and invasive ranges, with model selection performed using Akaike's information criteria. Genetic and environmental drivers that structured the life history of populations in their native range were less influential in their invasive populations. For some vertebrates at least, fitness‐related trait shifts do not seem to be dependent on the level of genetic diversity or haplogroup makeup of the initial introduced propagule, nor of the availability of local environmental conditions being similar to those experienced in their native range. As long as local conditions are not beyond the species physiological threshold, its local establishment and invasive potential are likely to be determined by local drivers, such as density‐dependent effects linked to resource availability or to local biotic resistance.

## Graphical abstract

Rapid adaptation to global change can counter vulnerability of species to population declines and extinction. Theoretically, under such circumstances both genetic variation and phenotypic plasticity can maintain population fitness, but empirical support for this is currently limited. Here, we found that genetic and environmental drivers that structured the life history of Pseudorasbora parva populations in their native range were less influential in their invasive populations.

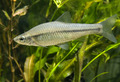

## 1 INTRODUCTION

Recent global environmental changes, and those predicted in the future, have added urgency to the quest to understand how natural populations respond to such challenges (Rands et al., 2010). Rapid adaptation to local environmental change can counter the vulnerability of species to population declines and extinction (Hoffmann & Sgro, 2011). Theoretically, under such circumstances both genetic variation and phenotypic plasticity can maintain the fitness of wild populations, but empirical support for this is currently limited due to the challenges involved in investigating the relationship between genetic variation and the role of phenotypic plasticity in maintaining fitness under novel and, often contrasting, environmental conditions (Ouborg, Pertoldi, Loeschcke, Bijlsma, & Hedrick, 2010).

Biological invasions provide a useful framework to study biological adaptation in natural settings as they represent a natural translocation experiment over multiple generations (Lowry et al., 2013). Environmental conditions often differ between the native and non‐native range of invasive species, with the challenge for a newly introduced species being to respond rapidly and efficiently to changes in the selective pressures imposed by the new ecosystem. Furthermore, a series of stochastic introduction events associated with the colonization process (Gozlan, Andreou, et al., 2010; Gozlan, Britton, Cowx, & Copp, 2010) is predicted to result in strong genetic drift and reduced genetic diversity in invasive populations balanced by epigenetic variations. Such low genetic burden is expected to limit the ability of the introduced species to establish invasive populations (Hanfling, 2007; Kelly, Muirhead, Heath, & Macisaac, 2006; Kolbe et al., 2004; Roman & Darling, 2007; Simon, Britton, Gozlan, van Oosterhout, & Hänfling, 2011). However, other studies contradict this and show that low genetic variation has no effect on the invasion success (e.g., Brown & Stepien, 2008; Planes & Lecaillon, 1998; Valiente, Ayllon, Nunez, Juanes, & Vazquez, 2010). Although many invasive species have low genetic diversity, some studies suggest that admixture of populations from genetically divergent sources could overcome genetic bottlenecks (Kelly et al., 2006; Kolbe et al., 2004). Nevertheless, it is currently not known whether such admixture, a by‐product of the invasion process, facilitates establishment (Estoup & Guillemaud, 2010). In addition, transcriptional plasticity plays an important role in adaptive responses. Hence, the invasive success might be reflected by epigenetic signatures regardless of the genetic diversity (Ardura, Zaiko, Morán, Planes, & Garcia‐Vazquez, 2017; Wellband & Heath, 2017). Thus, the introduction of a species into a non‐native habitat provides the opportunity for rapid evolutionary change through epigenetics, selection, and drift, and the majority of studies report marked phenotypic change in invasive populations (Bossdorf et al., 2005; Mooney & Cleland, 2001).

The study of life history traits (LHT) in the context of biological invasions is also central to understanding the process of local adaptation and is underpinned by life history theory (Kozlowski & Wiegert, 1986; Olden, Leroy Poff, & Bestgen, 2006; Roff, 1992; Stearns, 1992). LHT could help predicting how natural selection drives organisms to optimize their fitness (i.e., reproduction and survival) in light of environmental changes (Stearn, 2000). Such understanding is important as during invasions, adaptation is influenced by selection pressures in new abiotic and biotic conditions, but also may be constrained by the prior evolutionary history of the species and the stochastic bottlenecking of population genetic variation often associated with colonization (Keller & Taylor, 2008). Adaptation to new conditions, such as climate, can occur rapidly, facilitating rapid range expansion across the environmental gradients of the new range (Colautti & Barrett, 2013). Consequently, as other studies have shown (Brandner, Cerwenka, Schliewen, & Geist, 2013; Feiner, Aday, & Rice, 2012) it is expected that traits important for fitness will differ between native and non‐native environments, although it remains unclear whether the drivers of these traits important for fitness are influenced by the same abiotic/biotic pressures in both the native and the non‐native range. Indeed, biological traits that were useful in maintaining stable populations in the native range (e.g., K‐selected LHT such as low fecundity and slow growth) might lead a population to perform poorly in a new environment outside of their native range, where abundance may be low and where r‐selected traits (e.g., high fecundity and rapid somatic growth) would be more advantageous. Many studies have shown that LHT have a low level of heritability (Fox, Vila‐Gispert, & Copp, 2007; Price & Schluter, 1991; Schrieber et al., 2017) and that low heritability of LHT are theoretically expected to arise from high level of environmental variance (i.e., novel invaded habitat).

Here, we characterize the LHT of a model invasive species and quantify the relative roles of the environment, population genetic diversity, and prior evolutionary history during the invasion process. Three LHTs were assessed, including somatic growth rates, reproductive traits, and relative growth rates. The topmouth gudgeon *Pseudorasbora parva* is one of the most invasive fish in Europe that is now present in over thirty countries stretching from Eurasia to the most western part of Europe (Gozlan, Andreou, et al., 2010). It is regarded as one of the most damaging freshwater fish invaders in Europe due to their potential to have a devastating impact on native fish fauna through disease introduction (Al‐Shorbaji, Gozlan, Roche, Britton, & Andreou, 2015; Gozlan, St‐Hilaire, Feist, Martin, & Kent, 2005), and hence, research on this species has a high relevance to conservation ecology. Specifically, we test whether (a) LHT in the native range is associated with environmental parameters and/or haplotypes; (b) patterns of LHT and morphology in the native range are conserved across the invasive range; (c) the drivers of LHT in the invasive range remain the same as in the native range.

## 2 MATERIAL AND METHODS

### 2.1 Fish sampling and genetic data

The dataset comprised of 29 populations of *P. parva*: 17 from the native range in China, Japan, and Taiwan, and 12 from their invasive range in Europe, all collected between June and August 2010 (Table S1, Appendix S2). Following the sampling of each population (by fish trapping, seine netting or electric fishing, method dependent on the habitat sampled), 50 fish were randomly selected and euthanized (overdose of anesthetic, 120 mg/L benzocaïne), with fin tissues collected and preserved in 98% ethanol prior to the whole fish being preserved in 10% formalin. Each sampling site was geolocated using a GPS, and typology of the site recorded. Mean annual temperature (MTR) and rainfall (MRA) at the location of each sampled population were extracted from WorldClim database (Hijmans, Cameron, Parra, Jones, & Jarvis, 2005). Other climatic variables (Bio1‐Bio19) were tested in Fletcher, Gillingham, Britton, Blanchet, and Gozlan (2016) showing the importance of temperature and rainfall for this species.

For use in subsequent testing of its influence on life history trait expression, the genetic diversity of populations (GENDIV) in the dataset was available from Hardouin et al., (2018), where the genetic diversity of each population was based on mtDNA and microsatellite analysis conducted using the fin tissue samples (Table S1; Hardouin et al., 2018; Simon, Gozlan, Britton, van Oosterhout, & Hänfling, 2015). In addition, on mainland China there are two haplotypes present (Figure S1 in Appendix S1), one primarily located north of the River Yangtze (NH) and one south (SH) with the presence of admixed populations, and these represent the only two haplotypes introduced to Europe (Hardouin et al., 2018). Correspondingly, for each population the proportion of NH was also used as an explanatory variable in analyses of the life history trait data (percentage of NH: PNH).

### 2.2 Life history trait analyses

For each fish, fork length (FL, mm), weight (*W*, g), and sex were recorded. For the production of all data for somatic growth rate analyses, ages of individual fish were obtained from aging of scales, which were collected above the lateral line and below the insertion of the dorsal fin. Aging was completed on a projecting microscope by counting the number of annual growth checks present. Each fish length was then plotted against age (years), and the von Bertalanffy growth model parameter length infinity (Linf) and k (see Sainsbury, 1980) calculated. In addition, the growth metrics somatic growth rate (SGR (cm/year); FL_at capture_−FL_age 1_)/ age at capture) and FL at year one (FL1) were determined, which also corresponded to the age at maturity in all populations (Gozlan, Andreou, et al., 2010). All the growth parameters were calculated separately for males and females and averaged per population when appropriate. They were first all tested for correlations using the R package pairs (R Core Team, 2014). Locally weighted scatterplot smoothing (LOWESS) and Pearson correlation coefficient were calculated. General additive models were constructed to explain the distribution of the SGR data across the native and invasive ranges, with model selection performed using Akaike's information criteria following Burnham and Anderson's approach (2002; model.sel function with package MuMin, R Core Team, 2014). All of the models in the set of candidate models had exactly the same set of observations and therefore were based on the same sample size, along with the exact same response variables and the same method to calculate likelihoods. We chose, based on correlation values, a subset of models, which could be justified as good candidates for the best model on both statistical and biological grounds. Homogeneity of variance and normality of the data were checked for each model using QQ plots in the R’ computing program (R Core Team, 2014) to ensure model assumptions we not violated. Simple native versus non‐native comparisons of LHT were done using an independent 2‐group Mann–Whitney U test in R using the function wilcox.test.

Reproductive traits were assessed using fecundity and gonadosomatic index (GSI) of female fish. Ovaries were extracted from each female and weighed (to 0.01 g; Wo). A subsample of each ovary, cut from the middle of the gonad, was weighed (Ws), and the number of oocytes counted under a binocular microscope. Each oocyte was classified by size categories using a fitted micrometer (i.e., <0.1; [0.4–0.5]; [0.6–0.8]; [0.9–1]; >1.1 mm ± 0.05 mm) with the sum of oocytes (*n*) representing potential fecundity (oocytes < 0.1 mm were not included in counts). The potential fecundity was then calculated as [FEC = n*Wo/WS]. GSI was calculated as [Gonad weight/ Total fish weight] × 100 (Strum, 1978), and only females with GSI above 12% were considered in reproductive state and used in analyses. Female potential fecundity and SGR were initially plotted against each other for all populations (native and invasive), and their linear relationships at a population level analyzed using linear regression.

### 2.3 Relative growth (morphology)

To examine patterns of relative growth, raw data from 30 mensural characters, including fork length (FL; Table S2, Appendix S2; see also Záhorská et al., 2009), were measured from digital photographs taken by a Pentax optio S10 camera, with analysis using IMPOR 2.31E software. To examine patterns of relative growth, raw data from morphometric characters were plotted against FL, as described by Kováč, Copp, and Francis (1999) and size‐related variations (FL) among the populations were taken into account. Linear discriminant analysis (LDA) was applied to the morphological data of populations with pure haplotypes using the LDA from MASS R package. As it is a supervised technique (i.e., it uses class information), it provided a better data separation when compared to principal component analysis, while still presenting the possibility of dimensionality reduction, which is very useful for visualization. Prior to performing the LDA, the individual predictors were centered, scaled, and had skewness transformations applied, as per Kuhn and Johnson (2013). Thus, Box and Cox transformation (Box & Cox, 1964) was applied using the preprocess function from caret R package (Kuhn et al., 2016).

Wherever error around the mean is stated, it represents standard error.

## 3 RESULTS

### 3.1 Life history traits in the native versus invasive range

**Figure 1:**
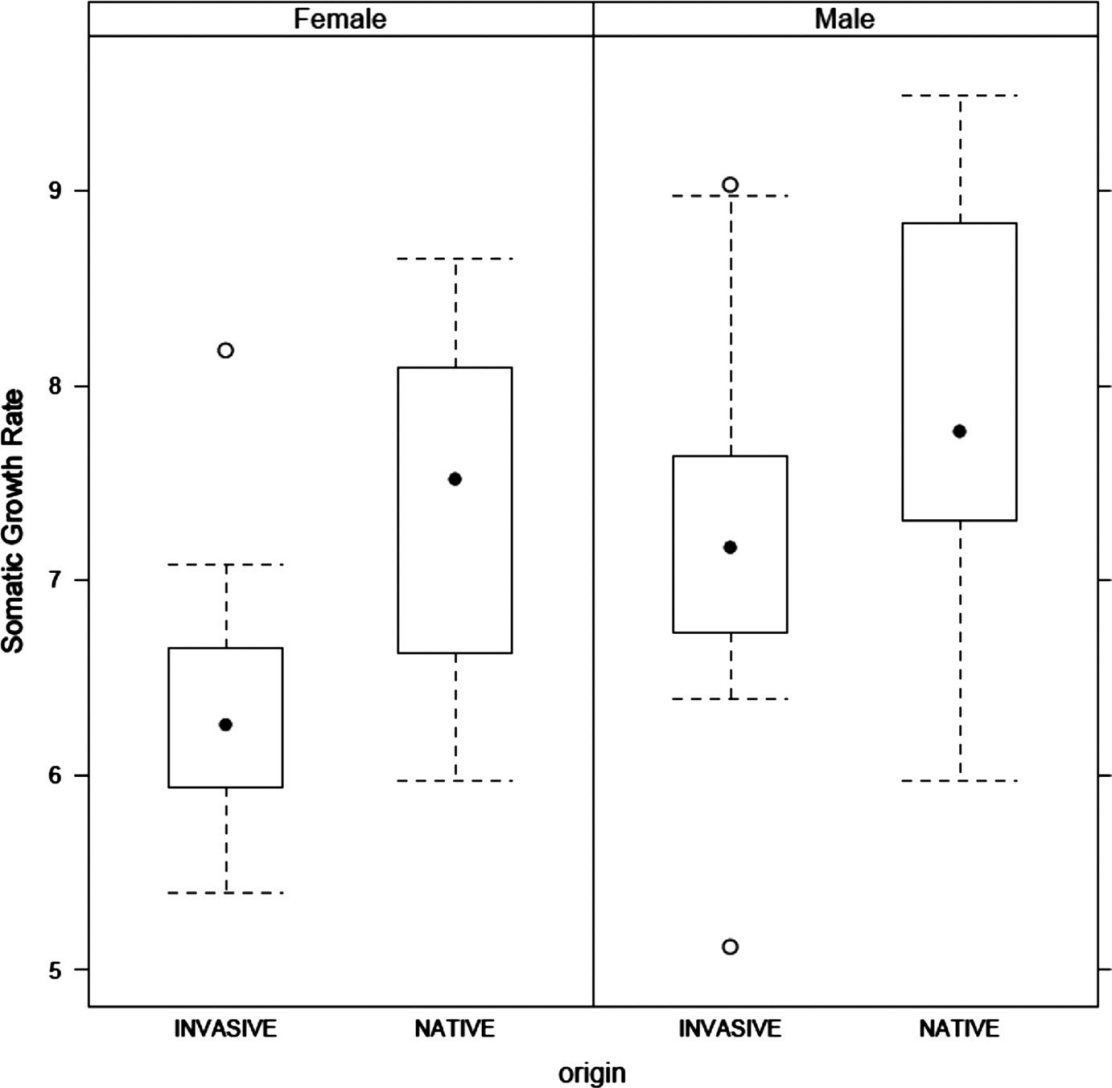
Boxplot of topmouth gudgeon *Pseudorasbora parva* somatic growth rate (SGR, cm/year) both for males and females across invasive and native populations (*n* = 25, see Table 1 for details)

**Figure 2:**
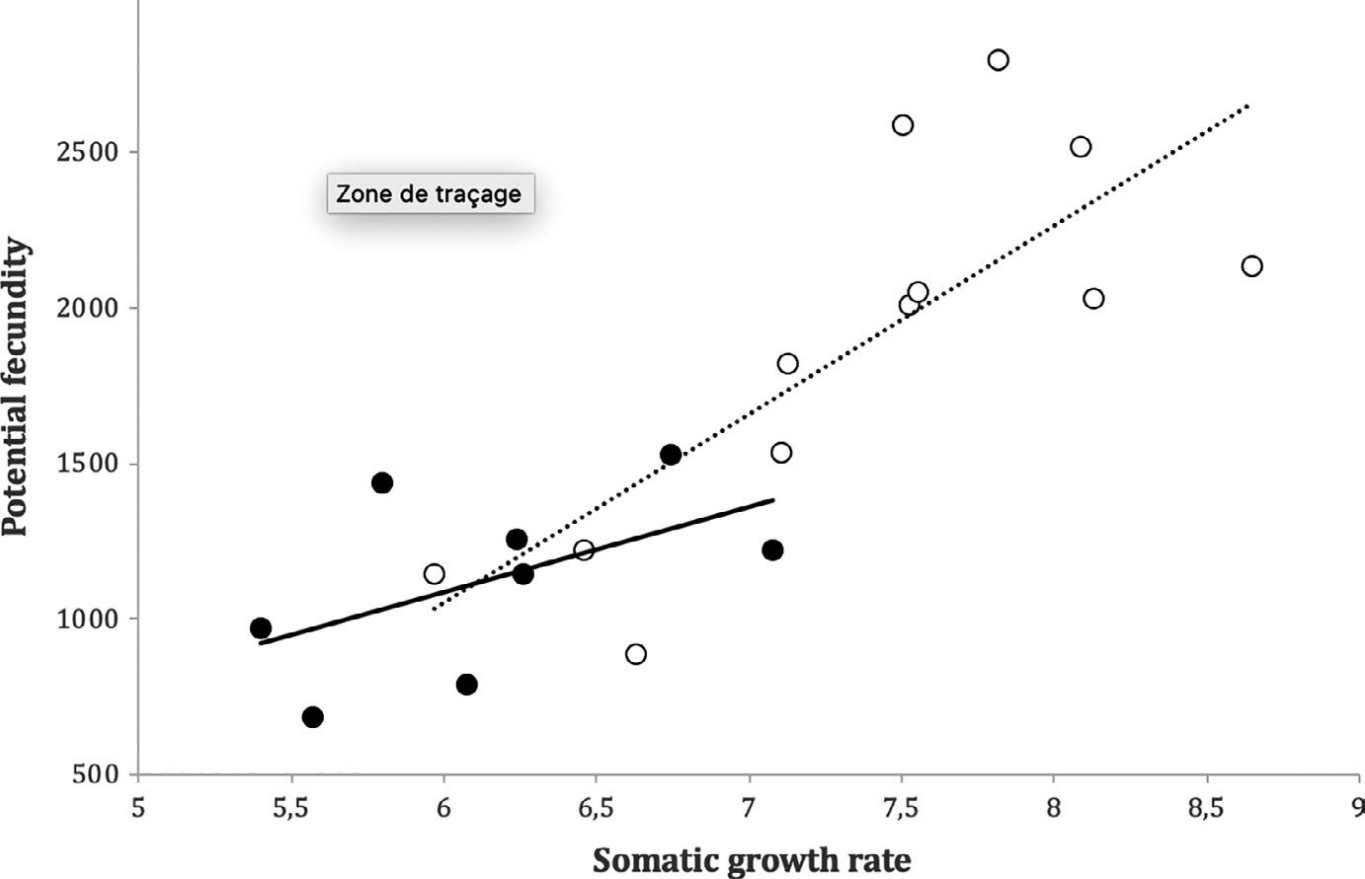
Potential fecundity of topmouth gudgeon *Pseudorasbora parva* across the native range (white circle) and the invasive range (black circle) in relation to the mean somatic growth rate (cm/year) of the population (*y*_invasive_ = 276.83*x* − 576.17, *R* ^2^ = .28 solid line; *y*_native_ = 605.37*x* − 2,578.5, *R* ^2^ = .60 dashed line)

Overall, females (NNF) in the invasive populations had significantly lower SGRs than native females (NF) (mean SGR_NF_: 7.35 ± 0.22 and mean SGR_NNF_: 6.40 ± 0.23, *W* = 32, *p* = .012), while males showed similar SGRs in both habitats (mean SGR_NM_: 7.84 ± 0.30 and mean SGR_NNM_: 7.26 ± 0.33, *W* = 54 *p* = .222; Figure 1). Also, females had significantly reduced fecundity in the invasive range (mean Fec_NF_: 1,890 ± 173 and mean Fec_NNF:_ 1,125 ± 105, *W* = 14, *p* = .009) (Figure 2). In addition, Linf and FL at year 1, surrogate for age at maturity in this species, were both correlated to the sex‐specific SGR regardless of the origin of the population (Figures S2 and S3 in Appendix S1). The fecundity of *P. parva* population was also significantly correlated to the SGR for native and non‐native populations combined (Figure 2). In effect, there was a loss of variability across all life history traits during the invasion process (see Figures 1 and 2) but also a loss of genetic diversity (GENDIV_N_: 0.67 ± 0.03; GENDIV_NN_: 0.55 ± 0.03, *W* = 36.5, *p* = .02).

### 3.2 Drivers of somatic growth

The general additive model revealed that in the native range, the mean annual temperature, and proportion of NH in the population explained 94.8% of the deviance of males SGR and 87.9% of the females SGR (see Tables 1 and 2). Also, rainfall patterns did not significantly influence the SGR of native populations (e.g., higher rainfall could lead to occasional flooding and be expected to negatively impact fish SGR through increased swimming expenditure), nor did population genetic diversity (Tables 1 and 2 and Figure S3). Among populations of the invasive range, neither temperature or rainfall (MTR, MRA) nor genetic variables (GENDIV, PNH) could significantly explain the SGRs for either sex (Table 1). Only 66.5% of the deviance for males SGR and 38.1% for females SGRs were explained by MTR and GENDIV (see Table 1 for summary statistics). However, it is important to bear in mind that mtDNA haplotypes (cytochrome b) as such do not necessarily have a causal influence on the measured LHT. It is likely that there are some other underlying factors that covary with these haplotypes, which could drive gene selection and local genetic adaptation. Thus, further genomic studies would be required if genetic adaptation processes were to be assessed postintroduction.

**Table 1:** Model selecting the best predictors of somatic growth rate using the protocol laid out by Burnham and Anderson (2002) between three general additive models for both males and females across native and invasive (gray) populations

	Int	MTR	GENDIV	PNH	MRA	*df*	logLik	AICc	Delta	Weight
ModF1	7.35	+		+		4	−1.92	16.3	0.00	0.802
ModF4	7.35	+		+	+	6	0.60	20.0	3.77	0.122
ModF2	7.35	+	+	+		5	−1.92	21.3	5.05	0.064
ModF0	7.35	+	+			4	−6.32	25.1	8.82	0.010
ModF3	7.35	+	+		+	6	−1.76	28.4	12.09	0.002
ModM1	7.85	+		+		6	−0.54	22.7	0.00	0.858
ModM2	7.85	+	+	+		9	18.45	27.1	4.49	0.091
ModM4	7.85	+		+	+	7	−0.27	28.7	6.06	0.042
ModM0	7.85	+	+			7	−1.71	32.1	9.40	0.008
ModM3	7.85	+	+		+	7	−3.50	35.7	13.05	0.001
ModM0	7.26	+	+			6	−10.24	49.1	0.00	0.998
ModM1	7.26	+		+		7	−2.87	62.9	13.73	0.001
ModM3	7.26	+	+		+	7	−9.29	63.5	14.36	0.001
ModM2	7.26	+	+	+		8	−2.76	97.8	48.65	0.000
ModM4	7.26	+		+	+	8	−2.90	100.1	50.96	0.000
ModF0	6.40	+	+			4	−9.63	33.9	0.00	0.454
ModF1	6.40	+		+		4	−9.65	34.0	0.04	0.445
ModF3	6.40	+	+		+	5	−8.28	38.6	4.65	0.044
ModF2	6.40	+	+	+		5	−8.34	38.7	4.76	0.042
ModF4	6.40	+		+	+	5	−9.34	40.7	6.76	0.015

Note

Adjusted Akaike's information criteria (AICc) are used to select the probability of being the best model within the set of models proposed. Parameters included are the genetic diversity from microsatellite (GENDIV), percentage of northern haplotype (PNH), the mean annual temperature (MTR), and the mean annual rainfall (MRA). Selected model performances are presented in Table 2.

**Table 2:** Approximate significance of smooth terms for the selected general additive models (see Table 1) within the native (white) and invasive populations (gray, *n*)

	*n*	edf	Ref.*df*	*F*	*p*‐value	$R_{\text{adj}}^{2}$	D_exp_	GCV	S_est_
ModM1						.86	87.9	0.12	0.09
Females									
MTR	14	1	1	25.121	**<.001**				
PNH	14	1	1	8.773	**.013**				
ModM1						.93	94.8	0.14	0.09
Males									
MTR	14	1.00	1.00	149.68	**<.001**				
PNH	14	2.66	2.92	5.21	**.021**				
ModM0						.42	66.5	1.13	0.65
Males									
MTR	11	2.66	2.91	2.28	.174				
GENDIV	11	1.00	1.00	0.48	.514				
ModF0						.23	38.1	0.64	0.46
Females									
MTR	11	1.00	1.00	2.251	.172				
GENDIV	11	1.00	1.00	0.072	.795				

Note

Parameters included are the genetic diversity estimated from microsatellites (GENDIV, Hardouin et al., 2018), percentage of northern haplotype (PNH), and the mean annual temperature (MTR). Degree of freedom (*df*) and effective degree of freedom are included (edf) as well as *F* and *p* values. Significant *p* values are in bold. The *R* square adjusted values ($R_{\text{adj}}^{2}$), the deviance explained (D_exp_), the generalized cross validation (GCV), and the scale estimate (S_est_) are included.

### 3.3 Morphological patterns

**Figure 3:**
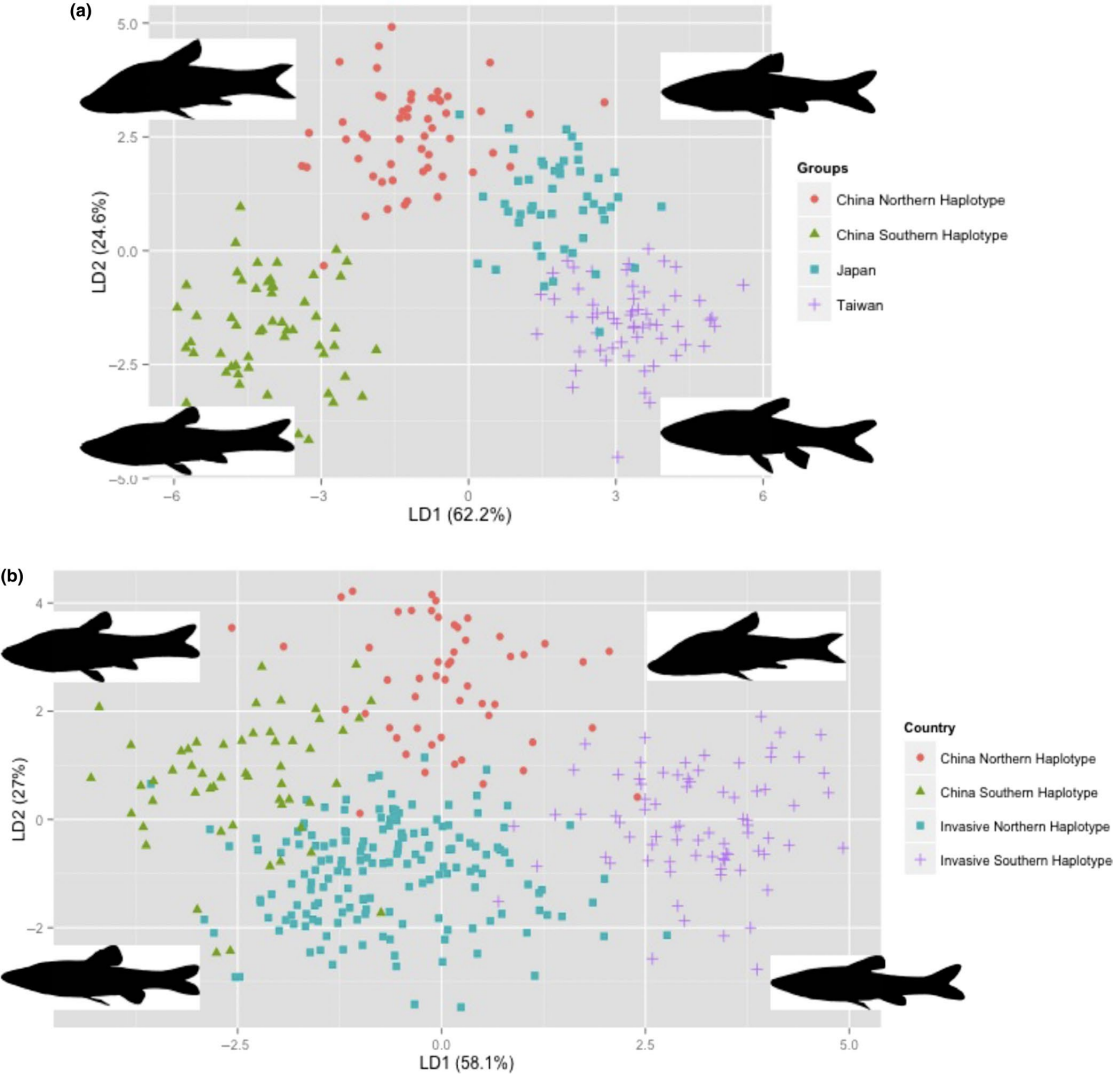
Linear discriminant analysis of topmouth gudgeon *Pseudorasbora parva's* morphology. (a) Comparison of the four native haplotypes and (b) comparison of the northern and southern Chinese haplotype in the native and the invasive range. The percentage for each LDA axis contribution is provided as percentages along each axis. See Table S2 (Appendix S2) for the list of the morphological traits measured and their coefficients of variation and Table S3 for overall fitness prediction

Linear discriminant analysis of the morphotypes associated with the pure haplogroups revealed a morphological separation between the two haplotypes found in mainland China, north (NH) and south (SH) of the Yangtze River (Figure 3a). It has also revealed that Japanese and Taiwanese haplogroups had a distinct morphology from the ones found in mainland China, albeit closer to NH than to SH. Finally, although the two mainland Chinese haplogroups retained separate morphologies during the invasion process, they also are both different to the original native morphologies (Figure 3b), drifting toward a dwarf morphotype as a result of their translocation into the invasive range but have not converged toward a common morphotype, with both NH and SH haplogroups retaining their morphological differences.

## 4 DISCUSSION

The results revealed that the genetic and environmental drivers that structured the life history of *P. parva* populations in their native range were less influential in their invasive populations. Somatic growth rates of both sexes among native populations were at least partly driven by mean annual temperatures and associated with the haplogroup structuring of populations (i.e., percentage of NH vs. SH; according to Hardouin et al. (2018) the NH and SH haplogroups separated about 2.5 million years ago). However, these two variables had limited influence on the somatic growth rates of invasive populations, indicating that although genetic and environmental variables were clear drivers of SGR of native populations, and this was not apparent in the invasive range, perhaps through being superseded by density‐dependent effects (Mueller, Guo, & Ayala, 1991). In the invasive populations, density‐dependent effects would typically rely on the local level of invasion (i.e., local population densities) and, as such, would not represent a consistent pattern across the whole invasive distribution range. Although we do not have the necessary data to test this hypothesis, it is supported by the experimental study of Britton and Gozlan (2013) that characterized the effect of propagule pressure on population and individual growth in *P. parva* and revealed highly variable growth rates between different stages of invasion.

Particular ecological drivers, such as predation or availability of food resources, could affect an organism's probability to survive and then reproduce following their introduction into a new range. Thus, understanding the drivers of SGR is central to characterize the optimal values and combinations of life history traits that maximize fitness, as SGR has a direct impact on female fecundity and female mating selection, where larger males are favored (Gozlan, Andreou, et al., 2010). Here, the outcome of SGR (and thus fecundity) across invasive populations contradicts existing studies on other invasive species (Chucholl, 2012; Fox & Copp, 2014; Hôrková & Kováč, 2014). For example, general patterns among animal invasive populations consistently reveal earlier maturity, maturity at a smaller size and greater reproductive investment, and thus show a more opportunistic suite of life history traits typical of r‐selected LHT (Hôrková & Kováč, 2014). Here, on the contrary, it is among native populations that we found a smaller size at maturity and higher fecundities, typical of r‐selected LHT. Based on the life history theory, such decreases in fecundity among invasive population could be explained by a greater offspring survival due to reduced competition and/or predation in the invaded environments, which correspond to the “equilibrium” life history strategy (late maturation, low batch fecundity, parental care, and high juvenile survivorship) defined by Winemiller and Rose, (1992). In addition, the loss of variability among LHT of invasive populations, although not surprising due to the accidental nature of *P. parva* invasion in Europe (i.e., low propagule pressure and reduced genetic diversity), may drive complex effects of intraspecific competition and lead to accelerated directional selection (Kelly et al., 2006; Kolbe et al., 2004). Thus, reduced fecundity, along with a reduced SGR, could simply result from limiting constraints and trade‐offs intrinsic to the *P. parva* invasive populations (Mueller et al., 1991). The enemy release hypothesis, which argues that introduced species are released of predators and pathogens, does not fit so well in the case of *P. parva* and could not strongly weigh in on the observed LHT patterns as (a) the species is also very abundant and invasive in the native range, (b) predation pressure (e.g., perch, pike, and trout are also high among invaded communities), and (c) the intensity of parasitism is similar across the native and invasive range (i.e., 19.4% prevalence in native range vs. 12.9% prevalence in non‐native range; Gozlan, Andreou, et al., 2010).

Alternatively, lower competition/predation pressures in the invasive range could be explained by the drastic impact of the highly virulent pathogen *Sphaerothecum destruens,* carried by *P. parva,* for local naive fish species (reviewed in Combe & Gozlan, 2018). Finally, such high fecundity, early maturity, and high SGR among native populations, in part, reflect the management of native wild fish populations in China, which has a long tradition of fish farming and freshwater fisheries. Such management maintains *P. parva* populations in a recurrent sate of high exploitation pressure, thus maintaining among native populations r‐selected LHT and high genetic variability via genetically admixed populations.

The morphology across the native range reflected the various haplogroups that have arisen from ancient segregation between Japan, Taiwan, and mainland China (Hardouin et al., 2018). It also supported the scenario of a southern colonization, as the mainland Chinese SH, which is also the youngest (Hardouin et al., 2018), and is morphologically the furthest away from those found in northern Chinese, Japanese, and Taiwanese populations. It is, however, unexpected that despite a convergence of the climatic niche experience by NH and SH during the European invasion (see Fletcher, 2018), and relatively low genetic diversity, no morphological convergence between these two haplogroups was observed. Both invasive NH and SH remained morphologically separated as well as having drifted away from native morphotypes. The presence of both morphologies but with individual of smaller sizes reveals dwarfism among invasive populations. This is a phenomenon which has been observed on islands (insular dwarfism), a process leading large individuals to have a reduced body size when their population's range is limited to a small environment, typical of ponds, lakes of *P. parva's* founder populations (Jordanaa & Köhler, 2011; Rozzi & Lomolino, 2017).

The approaches used to establish genetic patterns here (Hardouin et al., 2018) do not reflect gene selection and local genetic adaptation due to their neutral nature and thus do not directly underline the phenotypic plasticity of *P. parva*. Further epigenomic studies would then be required if adaptation processes and phenotypic plasticity were to be assessed postintroduction. Although epigenetics of freshwater invasions remains understudied, existing epigenomic variation studies have highlighted some interesting insights into the phenotypic plasticity and invasion status and success of non‐native populations (Ardura et al., 2017; Garcia et al., 2019; Wellband & Heath, 2017). The comparison of the epigenetic expression patterns of two invasive gobiidae species (Wellband & Heath, 2017) showed significant differences in the magnitudes and patterns of transcriptional changes between the more successful round goby and the less successful tubenose goby. Round goby transcriptional responses reflect alteration of biological function consistent with adaptive responses to maintain or regain homeostatic function while tubenose goby transcription patterns rather indicate a response to stressful conditions (Wellband & Heath, 2017). Hence, alterations to the epigenome may not only translate environmental changes into adaptive phenotypic responses but may also differentiate a successful invader from a less successful one. In the case of *P. parva*, based on the success of its invasion across Europe in such a short period of time, it seems to indicate transcriptional responses similar to the round goby.

## 5 CONCLUSION

The outputs of this study suggested that for some vertebrates at least, the process of trait shifts, although not necessarily driven by natural selection, does not seem too dependent on the genetic diversity or haplogroup makeup of the initial introduced propagule, nor of the availability of local environmental conditions being similar to those experienced in their native range. As long as local conditions are not beyond the species physiological threshold, its local establishment and invasive potential are likely to be determined by local drivers, such as density‐dependent effects linked to resource availability or to local biotic resistance (e.g., number of predators). Thus, understanding the drivers of LHT in the native range alone, as well as the native encountered climatic conditions, is of limited use to predict adaptability potential of the species and future invasions. Here, the epigenome analysis of native populations and introduced populations according to their invasion status (established, expanding, and equilibrium) would be of great added value. Furthermore, these results also indicate that species such as *P. parva* are unlikely to be affected by a change of climate as shown by Fletcher et al. (2016) and that despite a reduction in its gene pool, the species still displays a substantial ability to quickly adapt to major environmental changes and the effects of climate change.

## CONFLICT OF INTEREST

There is no conflict of interest. We declare that none of the authors listed on the manuscript are employed by a government agency that has a primary function other than research and/or education.

## AUTHOR CONTRIBUTION

**Rodolphe Elie Gozlan:** Conceptualization (lead); Data curation (lead); Formal analysis (lead); Funding acquisition (lead); Investigation (lead); Methodology (lead); Project administration (lead); Resources (equal); Validation (lead); Writing‐original draft (equal); Writing‐review & editing (equal). **Eva Zahorskae:** Methodology (equal); Writing‐original draft (equal); Writing‐review & editing (equal). **Emira CHERIF:** Conceptualization (equal); Writing‐original draft (equal); Writing‐review & editing (equal). **Takashi Asaeda:** Resources (equal); Writing‐original draft (equal); Writing‐review & editing (equal). **John Robert Britton:** Resources (equal); Writing‐review & editing (equal). **Cha‐Ho Chang:** Writing‐review & editing (equal). **To Hong:** Methodology (equal); Writing‐review & editing (equal). **Rafael Miranda:** Resources (equal); Writing‐review & editing (equal). **Jiri Musil:** Resources (equal); Writing‐review & editing (equal). **Meta Povz:** Resources (equal); Writing‐review & editing (equal). **Serhan Tarkan:** Resources (equal); Writing‐review & editing (equal). **Elena Tricarico:** Resources (equal); Writing‐review & editing (equal). **Teodora Trichkova:** Resources (equal); Writing‐review & editing (equal). **Hugo Verreycken:** Resources (equal); Writing‐review & editing (equal). **Andrej Weiperth:** Resources (equal); Writing‐review & editing (equal). **Andrej Witkowski:** Resources (equal); Writing‐review & editing (equal). **Lluis Zamora:** Resources (equal); Writing‐review & editing (equal). **Irene Zweimuller:** Resources (equal); Writing‐review & editing (equal). **Ya‐Hui Zhao:** Investigation (equal); Methodology (equal); Resources (equal); Writing‐review & editing (equal). **Hamid Reza Esmaeili:** Resources (equal); Writing‐review & editing (lead). **Marine Combe:** Conceptualization (equal); Validation (equal); Writing‐original draft (equal); Writing‐review & editing (equal).

## AUTHOR CONTRIBUTIONS

REG: Study design, performing initial research, and data analyses. REG, MC, HE, and EC: Drafting of manuscript. EZ: Morphological data acquisition. REG: Fecundity data. TH and JRB: Collection of age data from scales. REG, EZ, TA, JRB, CHC, TH, RM, JM, MP, AST, ET, TT, HV, AW, AW, LZ, IZ, YZ, and HE: Sampling populations. All authors contributed to the manuscript and approved it.

## Supporting information

Supplementary Material

## Data Availability

The data have been archive in a publicly accessible repository (Dryad https://doi.org/10.5061/dryad.m905qftz4).
