# Supplementary material for: Native drivers of fish life history traits are lost during the invasion process
Source: Ecol Evol. 2020 Aug 3;10(16):8623–33. doi: 10.1002/ece3.6521 (PMC7452779; doi:10.1002/ece3.6521)
Supplement: Supplementary file 1 — Supplementary Material [file ECE3-10-8623-s001.docx]

**Appendix S1** Mapping of the different haplogroups across Asia (native range) and Europe (invasive range), and locally weighted scatterplot smoothing as well as Pearson correlation for native and invasive populations. From Hardouin E.A., Andreou D., Zhao Y., Chevret P., Fletcher D.H., Britton J.R. and Gozlan R.E. (2018) Reconciling the biogeography of an invader through recent and historic genetic patterns: the case of topmouth gudgeon *Pseudorasbora parva*. Biological Invasion. 20 (8) 2157-2171

**Figure S1** Mitochondrial haplogroup distribution across *Pseudorasbora parva*’s geographical distribution: (A) native range (B) invasive range (see Hardouin et al. 2018 for full details). Two haplogroups are present on mainland China and have been introduced to Europe haplogroup (pink Northen & blue Southern). Abbreviations of locations can be found in Table 1. Figure 1: Summary of mitochondrial haplogroup and the structure distribution across Pseudorasbora parva’s geographical distribution: (A) native range, China, Taiwan and Japan and (B) Eurasia.


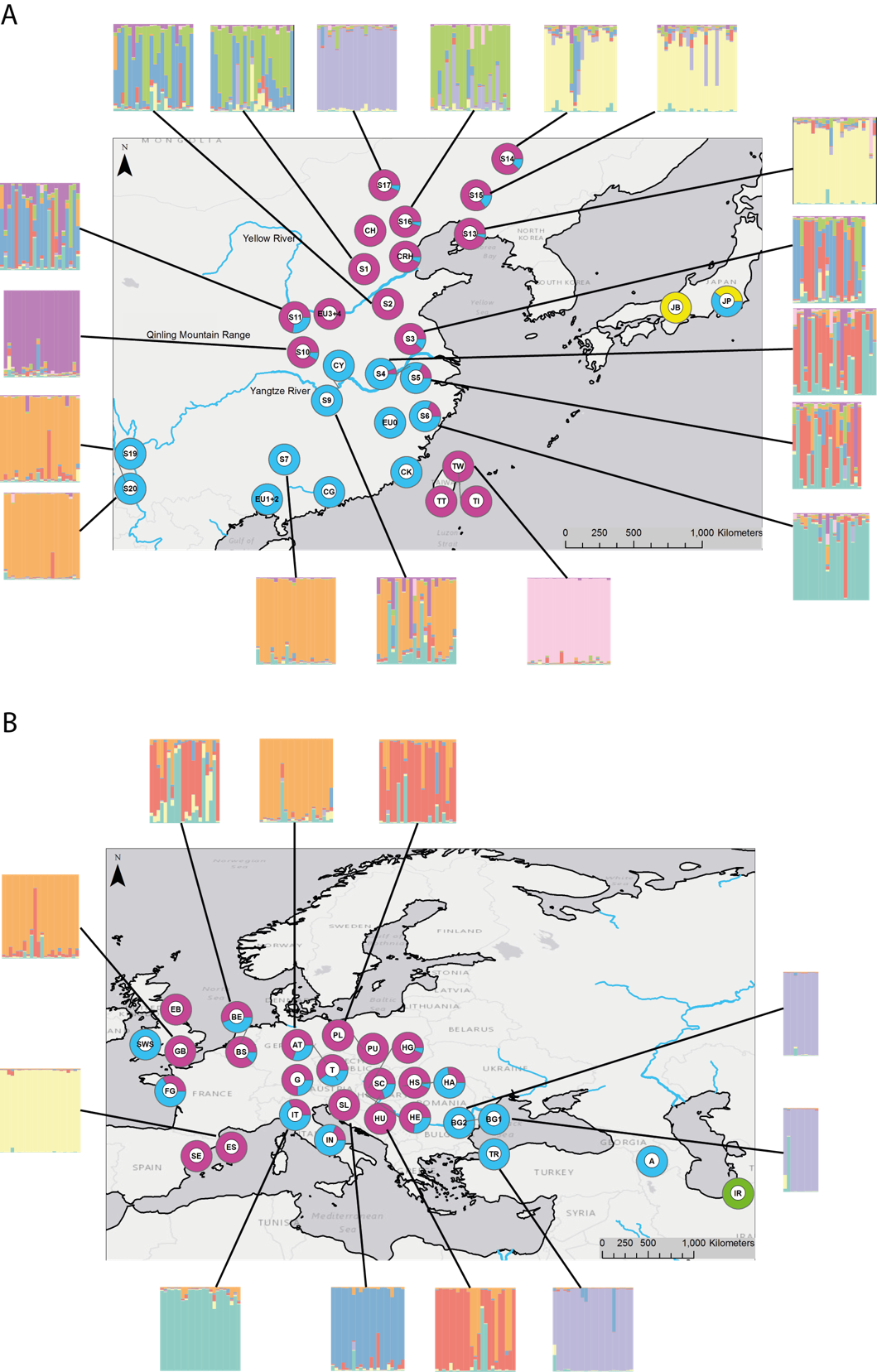


A

B

**Figure S2** Locally weighted scatterplot smoothing (LOWESS) and Pearson correlation coefficient matrix between a set of life history traits of topmouth gudgeon *Pseudorasbora parva* such as somatic growth rate (SGR), Von Bertanlanffy length at infinity (LIN), fork length at age one (FL1) for both male and females (M, F) across native populations as well as genetic parameters such as genetic diversity (GEDIV), percentage of northen haplotype and a couple of environmental variables such as the mean annual temperature (MTR) and rainfall (MRA).


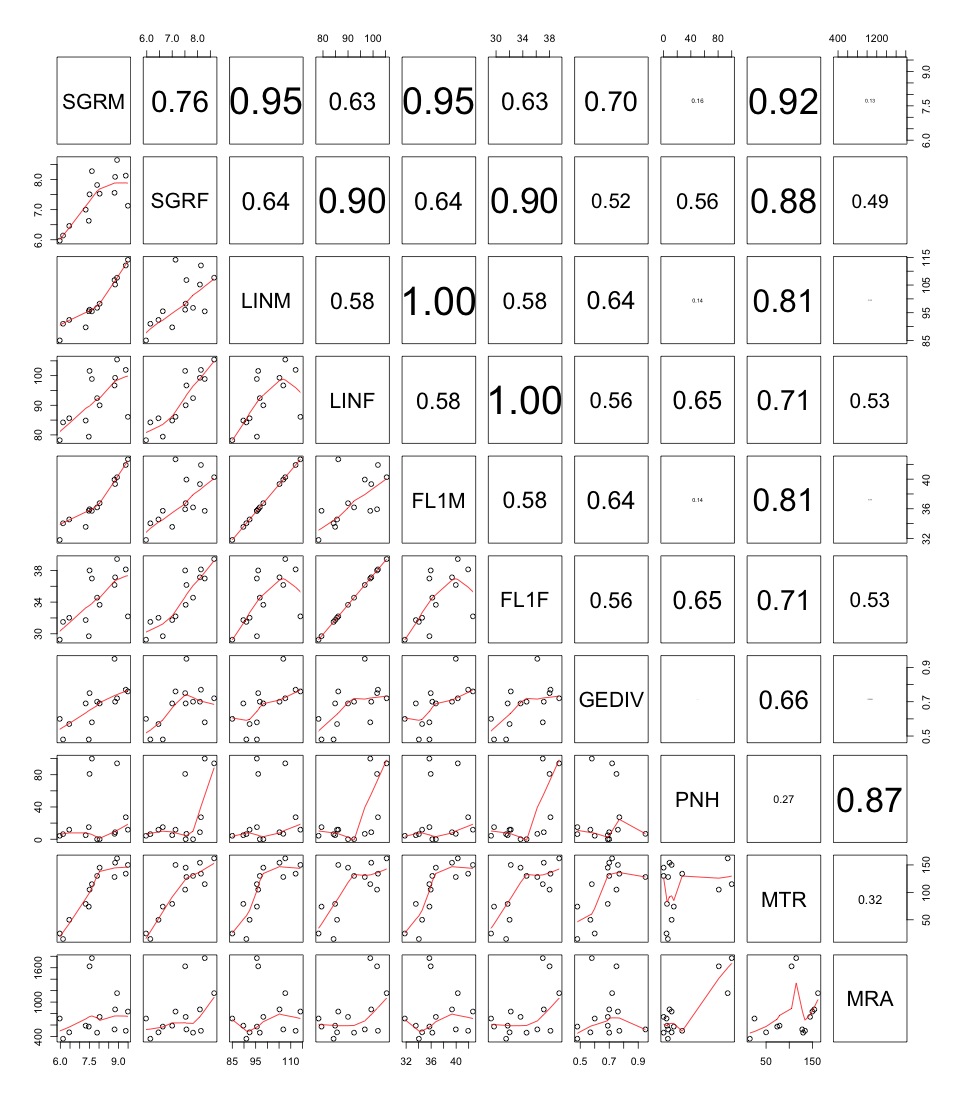


**Figure S3** Locally weighted scatterplot smoothing (LOWESS) and Pearson correlation coefficient matrix between a set of life history traits of topmouth gudgeon *Pseudorasbora parva* such as somatic growth rate (SGR), Von Bertanlanffy length at infinity (LIN), fork length at age one (FL1) for both male and females (M, F) across invasive populations as well as genetic parameters such as genetic diversity (GEDIV), percentage of northern haplotype and a couple of environmental variables such as the mean annual temperature (MTR) and rainfall (MRA).


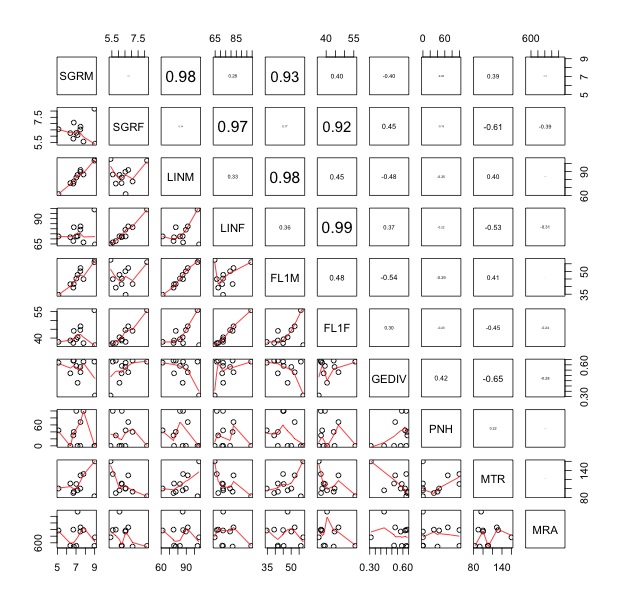


**Appendix S2** Topmouth gudgeon *Pseudorasbora parva* population parameters and locations for native and invasive populations used in this study, and list of the morphological traits measured.

**Table S1** Topmouth gudgeon *Pseudorasbora parva* population parameters and locations with invasive populations in grey. Parameters included are the genetic diversity from microsatellite (GEDIV, Hardouin *et al.,* 2018), percentage of northen haplotype (PNH), for both males and females length at infinity (LIN_F_ , LIN_M_), length at age 1 (FL1_F_, FL1_M_), somatic growth (SGR_F_, SGR_M_), mean potential fecundity (FEC). (MRA).

| Country |  | Latitude | Longitude | N | GEDIV | PNH | LIN_F_ | LIN_M_ | FL1_F_ | FL1_M_ | SGR_F_ | SGR_M_ | FEC |
| --- | --- | --- | --- | --- | --- | --- | --- | --- | --- | --- | --- | --- | --- |
| China | Pop1 | 37.55 | 115.56 | 23 | 0.70 | 100 | 92.41 | 96.76 | 34.56 | 36.187 | 7.82 | 7.90 | 2791 |
|  | Pop2 | 34.81 | 117.12 | 23 | 0.69 | 100 | 90.00 | 98.27 | 33.66 | 36.754 | 7.53 | 8.03 | 2005 |
|  | Pop3 | 33.19 | 118.59 | 21 | 0.76 | 88.2 | 86.10 | 114.13 | 32.2 | 42.683 | 7.13 | 9.49 | 1819 |
|  | Pop4 | 31.40 | 118.57 | 23 | 0.72 | 5.9 | 105.44 | 107.65 | 39.436 | 40.262 | 8.65 | 8.93 | 2129 |
|  | Pop5 | 28.12 | 119.57 | 21 | 0.75 | 17.6 | 88.94 | 104.07 | 33.264 | 38.924 | 6.74 | 8.54 |  |
|  | Pop6 | 25.27 | 110.32 | 22 | 0.58 | 19 | 101.60 | 96.08 | 37.998 | 35.934 | 7.51 | 7.51 | 2582 |
|  | Pop7 | 32.56 | 111.55 | 23 | 0.70 | 0 | 98.88 | 95.50 | 36.982 | 35.718 | 8.28 | 7.63 |  |
|  | Pop9 | 40.10 | 122.52 | 23 | 0.60 | 0 | 80.64 | 102.15 | 30.16 | 38.204 | 5.96 | 8.64 |  |
|  | Pop10 | 45.03 | 124.99 | 22 | 0.57 | 65.2 | 99.28 | 105.18 | 37.131 | 39.337 | 8.09 | 8.83 | 2515 |
|  | Pop11 | 42.64 | 122.93 | 23 | 0.48 | 68.2 | 101.95 | 112.08 | 38.13 | 41.917 | 8.13 | 9.38 | 2024 |
|  | Pop12 | 40.90 | 118.27 | 23 | 0.69 |  | 96.70 | 106.80 | 36.166 | 39.943 | 7.56 | 8.80 | 2048 |
|  | Pop13 | 43.30 | 116.89 | 23 | 0.48 | 95.7 | 78.22 | 84.99 | 29.253 | 31.788 | 5.97 | 5.97 | 1144 |
|  | Pop14 | 23.56 | 99.94 | 23 | 0.65 | 88.2 | 85.62 | 92.40 | 32.023 | 34.558 | 6.46 | 6.46 | 1215 |
|  | Pop15 | 23.35 | 99.53 | 22 | 0.54 | 85 | 79.41 | 95.53 | 29.698 | 35.728 | 6.63 | 7.47 | 885 |
| Japan |  | 139.43 | 35.67 | 23 | 0.76 | 0 | 75.12 | 81.90 | 28.096 | 30.631 | 6.48 | 6.48 |  |
| Taiwan |  | 136.07 | 35.25 | 23 | 0.40 | 0 | 81.90 | 103.56 | 30.629 | 38.733 | 7.11 | 9.68 | 1531 |
| Austria |  | 48.19 | 14.72 | 21 | 0.64 | 70 | 67.88 | 74.77 | 37.21 | 39.3 | 5.8 | 6.71 | 1437 |
| Belgium |  | 50.94 | 4.80 | 20 | 0.62 | 55.6 | 72.48 | 61.7 | 37.82 | 34.62 | 6.55 | 5.12 |  |
| Bulgaria |  | 44.06 | 26.85 | 10 | 0.59 | 0 | 72.43 | 82.44 | 39.34 | 45.28 | 6.26 | 7.03 | 1144 |
| Czech R |  | 14.18 | 49.15 | 25 |  | 60 | 81.52 | 77.44 | 44.09 | 41.68 | 7.08 | 6.76 |  |
| England |  | 51.00 | -1.45 | 22 | 0.58 | 100 | 79.1 | 89.54 | 44.55 | 50.08 | 6.53 | 7.46 |  |
| Hungary |  | 46.63 | 18.87 | 23 | 0.52 | 100 | 71.65 | 75.38 | 38.63 | 41.59 | 6.24 | 6.39 | 1251 |
| Iran |  | 37.05 | 54.78 |  | 0.54 | 0 | 74.48 | 80.91 | 39.18 | 43.36 | 6.67 | 7.1 |  |
| Italy |  | 44.77 | 10.52 | 23 | 0.53 | 31.8 | 82.37 | 91.79 | 46.68 | 52.21 | 6.75 | 7.48 | 1526 |
| Poland |  | 51.19 | 17.19 | 22 | 0.63 | 100 | 99.04 | 103.5 | 55.8 | 56.09 | 8.18 | 8.97 |  |
| Slovenia |  | 15.33 | 15.33 | 21 | 0.43 | 100 | 72.6 | 85.71 | 40.47 | 47.76 | 6.08 | 7.17 | 785 |
| Spain |  | 41.57 | 2.53 | 23 | 0.31 | 100 | 64.55 | 105.32 | 35.99 | 57.57 | 5.4 | 9.03 | 968 |
| Turkey |  | 40.91 | 30.04 | 23 | 0.63 | 0 | 66.57 | 86.18 | 37.11 | 44.91 | 5.57 | 7.8 | 679 |

**Table S2** List of topmouth gudgeon *Pseudorasbora parva*’s morphological traits (n= 27) measured according to Záhorská et al. 2009 in addition to standard, fork and total length. Coefficients of linear discriminants are also provided for each variable of Figure 3a &b.

| **Morphological traits** | **LD1 (a)** | **LD2 (a)** | **LD1 (b)** | **LD2 (b)** |
| --- | --- | --- | --- | --- |
| Head length  Pre-orbital distance  Eye diameter  Post-orbital distance  Head depth  Pre-dorsal distance  Pre-ventral distance  Pre-anal distance  Pectoral-ventral distance  Ventral-anal distance  Body depth  Dorsal-Anal fin distance (anterior end)  Dorsal-Anal fin distance (posterior end)  Caudal peduncle length (dorsal)  Caudal peduncle length (ventral)  Post-dorsal distance  Post-anal distance  Dorsal fin base length  Anal fin base length  Pectoral fin length  Ventral fin length  Caudal upper lobe  Caudal lower lobe  Dorsal fin length  Anal fin length  Caudal peduncle  Gape | 49.6230914  -87.2660341  65.7747665  -56.4757384  -4.9766416  -9.1577567-11.2947688  -0.6867786  11.5627670  9.2389956  41.2205446  -32.0343042  -25.0975642  -17.6999516  19.4824891  51.3087301  -29.9232304  9.0050726  21.3159606  -10.4512178  -13.6292213  24.4105017  1.2692808  -6.2257964  -3.1939263  55.5643283  63.8054335 | 7.0885914  57.3885043  -70.6361835  -35.7836457  13.1751429  4.9936061  -12.0109929  7.7822003  13.5263434  -15.7837457  -4.4887069  -22.6701490  55.6654527  -2.6296851  34.9231766  19.2589589  -37.1443145  49.1636422  24.8019835  -38.3253602  -4.1291061  -2.5585473  0.7990032  -13.6006489  -13.7186072  30.3857742  10.4043852 | 21.3213372  -6.3854400  68.6948027  -25.4414306  -17.5523265  -15.4847362  -20.4672015  -0.9255329  -4.2358603  1.2488437  40.7995581  -5.9735539  -48.9740573  -4.2104177  5.2560726  32.6972998  2.2269051  -8.5656756  -3.1345073  -11.5374272  -22.1899035  8.0169670  4.8890466  -7.9290151  -14.0335728  33.2419090  58.5651160 | 9.2592179  93.5797781  -50.3070521  -9.2156233  11.4821967  13.7785854  -21.5013335  0.0356474  6.1598189  -3.1815038  32.7664665  -16.8184208  9.4317319  2.1908116  10.8750012  8.7541778  6.5149954  27.6658021  9.2249627  -19.9066454  -5.7601478  -7.6400955  -5.3167891  2.9782442  -34.8518134  -74.2578331  -54.0590633 |

**Table S3** Overall predictive fitness of the LDA model showing a relatively good level of prediction. A **=** China Northern Haplotype; B = China Southern Haplotype; C = Invasive Northern Haplotype; D = Invasive Southern Haplotype

|  | **A** | **B** | **C** | **D** |
| --- | --- | --- | --- | --- |
| **A** | **47** | 3 | 0 | 0 |
| **B** | 2 | **48** | 1 | 0 |
| **C** | 3 | 3 | **162** | 4 |
| **D** | 1 | 0 | **5** | **74** |
